# Bioremediation of Pharmaceuticals, Pesticides, and Petrochemicals with Gomeya/Cow Dung

**DOI:** 10.5402/2011/362459

**Published:** 2011-04-26

**Authors:** Gurpreet Kaur Randhawa, Jagdev Singh Kullar

**Affiliations:** ^1^Department of Pharmacology, Government Medical College, Amritsar, Punjab 143001, India; ^2^Department of Anatomy, Government Medical College, Amritsar, Punjab 143001, India

## Abstract

Use and misuse of pharmaceuticals, pesticides, and petrochemicals by man is causing havoc with nature, as they persist as such or as their toxic metabolites. These pollutants bioaccumulate in environment, and they ultimately reach man through various means. They are hazardous because of potential toxicity, mutagenicity, carcinogenicity, and genotoxicity. To rejuvenate nature, remediation methods currently available are usually expensive and might convert one toxic pollutant to another. Bioremediation methods use naturally occurring microorganisms to detoxify man-made pollutants so that they change pollutants to innocuous products that make soil fertile in the process. Taking cue from Ayurveda, Gomeya/cow dung is used as an excellent bioremediation method. Thus, utilizing freely available cow dung as slurry or after composting in rural areas, is a cheap and effective measure to bioremediate the harmful pollutants. Yet, more research in this direction is warranted to bioremediate nonbiodegradable, potentially toxic pollutants.

## 1. Introduction

Synthetic and semisynthetic pharmaceuticals and pesticides are known to pollute the aquatic, terrestrial, and atmospheric environment alike, and they usually find their way into the drinking water as a dilute cocktail of varied drugs in varied concentrations. About 26 metric tons of pharmaceutical waste is disposed annually down the drain, and another 26 tons are disposed annually with municipal solid waste in landfills in North America alone [1]. North American [2], Canadian [3], Japanese [4], Korean [5], and across the Europe, waterways [6] contain traces (in nanograms/L to micrograms/L) of antibiotics, painkillers, hormones, tranquilizers, anti-inflammatory, chemotherapeutic, antiepileptic and hypolipidemic drugs [7–11]. Pesticide residues are also above the permissible levels. The persistence of organo-xenobiotics in the environment is a matter of significant public, scientific, and regulatory concern because of the potential toxicity, mutagenicity, carcinogenicity, and genotoxicity. These pollutants tend to effect the ecosystem in a negative way. 

Nonbiodegradable, synthetic and semisynthetic compounds find their way into the environment either through excretion after human use, agriculture and veterinary use, petrochemical waste, through disposal of expired medicine, and as a manufacturing waste. Biodegradation aspect of new drug entities is not a priority with the pharmaceutical companies when they manufacture more efficacious drugs for various diseases. The medicaments with specially devised pharmacokinetic parameters conducive with a long duration of action within the body are the ones which also persist in the environment for a longer time. Long duration of action can be achieved by chemical modifications in the structure of drugs, manufacturing-extended, contin, or delayed release preparations. They tend to bioaccumulate and travel up in the food chain and can affect humans directly or indirectly. Their accumulation in environment also poses risk to other nontargeted organisms, animals and humans.

## 2. Effect of Consuming a Dilute Cocktail of Synthetic Substances?

Select chemical/medicinal combinations can exhibit additive or synergistic toxic effects [12–14], and even compounds with different mechanisms of action can have interactive toxicological effects [15]. Many times, the wanted effects of medicaments in target species are the adverse effects in nontargeted species. In targeted species, effect of monotherapy/combination therapy for a specific time is well intended and documented. In non-targeted species, a cocktail of medicines in different combinations and permutations are chronically ingested unintentionally over prolonged periods of time. Their chronic cumulative impact on various types of organisms at different trophic levels has not been studied in detail though theoretically, it can be disastrous. Even though individual concentrations of any drug might be low, the combined concentrations from drugs sharing a common mechanism of action could be substantial. The primary pharmacodynamic activities of drugs in humans could induce effects totally different from the therapeutic ones in nonmammalian organisms [16]. Many times, the change is subtle like in terms of change in behavior, whose documentation is often difficult. The increased incidence of various carcinomas, thyroid and gastrointestinal tract diseases, and psychiatric illnesses can be sequelae to chronic exposure to ecodamaging chemicals in the nature. 

The results of three European research projects (ERAVMIS, REMPHARMAWATER, and POSEIDON) have established the environmental impact of both human and veterinary antibiotics, and it has been tabulated in Table 1 [17, 18].

There is some evidence that many of these substances of pharmaceutical origin are not degraded by sewage treatment plants (STP) and are also not biodegradable in the natural environment [11, 19, 20]. In the STPs, removal rates of pharmaceuticals vary from 0% (Carbamazepine, clarithromycin, erythromycin, estrone, lincomycin, and spiramycin) to 30%–60% (amoxycillin, ciprofloxacin, enalapril, ibuprofen, ofloxacin, phenytoin, 5-fluorouracil, and diclofenac) only [21]. 

*Rejuvenation of Environment* is a task of utmost importance and employing physicochemical processes only transform the pollutants from one form to another but biological processes transform them into harmless, innocuous end products. These concerns continue to drive the need for the development and application of viable and low-cost remediation techniques on large scale. Bioremediation is one such technology that offers the possibility to destroy or render harmless various contaminants using natural biological activity often by utilizing locally available constituents from the farms. Therefore, the need of the hour is to look for options in our (India's) thousands-of-year's-old (~1200 years old) Vedic literature, and how our ancestors were able to preserve Mother Earth for us, and in the process were able to lead a healthy life till ripe old age.

## 3. Bioremediation

Bioremediation is the use of naturally occurring microorganisms or genetically engineered microorganisms (bacteria and fungi) by man, to detoxify man-made pollutants [22]. Earthworms are capable of bioaccumulating heavy metals in their body tissues especially chloragocytes, and their intestinal microflora has the capacity to detoxify most of the pesticides. Earthworms are good additions to enhance the activity of natural and cheap composts to detoxify the environment. Microorganisms have a unique ability to interact both chemically and physically with a huge range of both man-made and naturally occurring compounds leading to a structural change to, or the complete degradation of the, target molecule. 

### 3.1. Vedic Literature

*Ayurveda *is one of the life sciences of the Vedic Indian period. Panchgavya Chikitsa is a part of Ayurveda (i.e., therapy with cow products, namely, milk, curd, clarified butter, urine, and dung). It is one of the main principles is that the world is made up of a combination of the five basic elements—ether, earth, air, water, and fire with a harmonious blend in the human body, flora, and fauna alike.

Susruta (one of the pioneers of Ayurveda) mentions that the human body is made up of these five basic elements in a delicate balance—Asimnchhaste panchmahabhut sharire samvaiym purusheh etyuchyte.22 (Sushruta Samhita 1 [23])

Susruta also says that when this delicate harmonious balance is interfered with, there would be disease in the world. Bhutebhyo hi param kinchinnasti chinta chikitsite.8 (Sushruta Samhita 1 [23])

Nature has made various natural mechanisms by which all waste are biodegraded naturally leaving no toxins in the environment, thus they do not harm the environment in any manner.

## 4. Gomeya/Cow Dung

According to Ayurveda, Gomeya/cow dung is not a waste product, but it is a purifier of all wastes in the nature [24]. When spread over urban and rural waste in solution form (1:10–1:25 solution), it biodegrades the waste in time. It is a “gold mine” due its wide applications in the field of agriculture, energy resource, environmental protection, and therapeutic applications. 

Cow dung is a cheap and easily available rich source of microflora. Though cow dung has been used in several studies, but the breed of cow has not been mentioned. As per Indian Vedic scriptures, cow dung obtained from Indian indigenous cow/*Bos indicus/Zebu* breed is better than that of other newer breeds. Ideally, the source of cow dung as per Ayurveda should be from a healthy Zebu cow, fed upon healthy diet of pastures including various natural herbs and which has been reared hygienically.

Shranivartan vrtya ne nivartan vrtya Bhumyachshrtrsm prdishstabhyam ena ne vrtya (Rigveda 10, 19, 8 [25])

Rigveda advises man to let the cows graze freely in the meadows in all the four directions everyday without any restrictions.

## 5. Composition of Gomeya

It is a mixture of dung and urine in a ratio of around 3 : 1. It contains crude fibre (cellulose with lignin), crude protein, cellulose, hemicellulose, and 24 minerals like nitrogen, potassium, traces of sulphur, iron, magnesium, calcium, cobalt, manganese, and so forth [26]. Microbial composition of cow dung includes about 60 species of bacteria (Bacillus species, corynebacterium species, and lactobacillus species), fungi (aspergillus and trichoderma), about 100 species of protozoa and yeasts (saccharomyces and candida). Majority of bacteria are cellulose, hemicelluloses, and pectin fermenters. Cow dung comprises of undigested fibre, sloughed off intestinal epithelium, some excreted products derived from bile (pigments), intestinal bacteria, and mucus. The bile pigment biliverdin is mainly present in cow dung (herbivore) giving it its green color. Bile salts give dung its emulsifying properties by conferring hydrophilic coat to otherwise hydrophobic droplets.

*Gomeya/cow dung slurry* usually a ratio of 1 : 10 or 1:25 is sprinkled over rural, urban and hospital waste, and oil spillage to degrade them naturally into the basic five elements. Cow dung slurry contains bacteria, fungi, and actinomycetes, namely, *Fecal streptococcus, Streptococcus, Pseudomonas* sp.,* Sarcina, Nocardia, Mucor* spp.*, Phizopus stolonifer, Rhizopus* sp.*, Aspergillus, E. coli* sp., and* Penicillium *microbes [27].

*Composting* (Bioaugmentation) is one of the bioremediation strategies which when carried out under controlled conditions in the presence of oxygen results in the biological decomposition and stabilization of the biodegradable components. The process of composting includes four main phases, which are the initial phase, the thermophilic phase, the mesophilic phase, and the maturation phase after which the compost can be used as an organic amendment. The degree of organic matter humification is generally accepted as a criterion of maturity or stabilization of compost. The humification process produces functional groups, and so increased oxidation of the organic matter leads to rise in cation exchange capacity. So, compost with high cation exchange capacity is regarded as an index of maturity. The degree of maturity can also be revealed by biological methods involving seed germination and root length, since immature composts may contain phytotoxic substances such as phenolic acids and volatile fatty acids. 

Composting is now increasingly used to accelerate the breakdown and transformation of pollutants including pesticides and for the stabilization of heavy metals in soil. These interactions ensure that pollutants are exposed to a broad range of microbes in the environment thereby increasing chances of their breakdown or transformation by different microbes. Additionally, some organic compounds formed during composting can bind some metals in ways that prevent their easy removal and thus their translocation from sensitive ecosystems.

In another study [28], cows of cross bred and indigenous/desi breeds were fed similar feed for 21 days, and thereafter, dung was collected for 6 days and analyzed for the organic and mineral content as shown in Table 2. 

## 6. Bioremediation of Pharmaceuticals and Pesticides

### 6.1. Antimicrobial Agents

In medicine, 6% of prescriptions are for antimicrobial agents, while in veterinary medicine, more than 70% of prescriptions contains them [29]. Therefore, nondegradation of antibiotics can theoretically lead to the development of multidrug resistant strains which can indirectly infect the humans, causing increased morbidity and mortality. 

Manure (e.g., cattle dung) could serve as a relevant model ecosystem to study the fate of drugs, more so because some of the known coprophilous basidiomycetes can degrade enrofloxacin. Two such basidiomycetes were isolated from aged cattle dung by Wicklow and colleagues two decades ago [30, 31]. Both isolates, strain NRRL 6464 and a strain identified as Cyathus stercoreus, showed high activity in the degradation of lignocellulose in vitro. C. stercoreus is able to degrade enrofloxacin [31, 32]. The bioremediation of some antimicrobial agents have been discussed by some researchers and is given in Table 3.

### 6.2. Biomedical Waste Degrader

Proper and cheap method of disposal of biomedical waste is a burning issue in view of the expanding health care system in India. Current method of biomedical waste disposal is the use of incinerators which are not only expensive but also not environment friendly. Incinerators produce toxic gases (polychlorinated dibenzofurans and polychlorinated dibenzo p-dioxins) in the process. Dioxins are known to cause genetic aberrations, hormonal imbalances, and damage to immune and reproductive systems. *Periconiella* species of fungus isolated from cow dung was found to be an excellent degrader of biomedical waste. Fifty grams of biomedical waste, kept in the form of used bandages and cotton in culture media, were effectively and completely reduced by 50th day. It was found to be cheap, safe, and environment friendly method of biomedical waste disposal [37].

### 6.3. Pesticides

At present, India is the largest producer of pesticides in Asia. The Indian Pesticide Industry with 82000 MT of production for the year 2005-2006 is ranked second in Asia (behind China) and ranks twelfth in the world for the use of pesticides with an annual production of 90,000 tons [38]. 

2%-3% of pesticide is actually utilized and the rest persists in soil and water causing environmental pollution leading to toxicity [39]. Pesticide residues remain in surface soil, leading to toxicity in the soil water environment. A vast majority of Indian population (56.7 percent) is engaged in agriculture and is, therefore, exposed to the pesticides used in agriculture. 

At present, the pesticide waste is being treated by physico-chemical methods which are not efficient and effective. As a result, pesticide residue remains in the soil-water environment causing toxicity to the biota and thereby entering into the food chain. Pesticide residues in animal products, and other food items ultimately get accumulated in man especially in the adipose tissue, blood, and lymphoid organs. 

Pesticide residue in environment ultimately affects the health of man and is a cause of morbidity. Immunopathological effects of pesticides in animals and man are acquired immunodeficiency or immunosuppression, and autoimmunity and hypersensitivity reactions, like eczema, dermatitis, allergic respiratory diseases, and pesticides might be the cause of recurrent infections. Many pesticides are known to cause mutations in chromosomes of man and animals, thereby may lead to carcinoma of liver and lung. They are teratogenic and mutagenic in nature, and can cause neuropathy, nephropathy, hepatotoxicity, and reproductive disorders [40].

In a study, pesticides (chlorpyrifos, cypermethrin, fenvalerate, and trichlopyr butoxyethyl ester) were analyzed for bioremediation with cow dung (specific breed of cow not mentioned) slurry. Fresh cow-dung slurry in the ratio of 1 : 10 with distilled water was taken as a source of microbial biomass. The cow dung slurry biomass was activated for a period of three days by continuous aeration and by addition of one dose of nutrient-glucose 150 mg/L, potassium dihydrogen phosphate 80 mg/L and ammonium sulphate 80 mg/L. Chlorpyrifos was rapidly hydrolyzed to 3,5,6 trichloro-2-pyridinol (TCP) in 25 and 50 mg/kg chlorpyrifos amended soil, while in 100 mg/kg chlorpyrifos amended soil, it was present till the 3rd day of the experiment. More than 75% and 50% Cypermethrin (25 and 50 mg/L, resp.) was hydrolyzed to 3-phenoxy benzaldehyde and 3-phenoxy-benzyl alcohol by 7th day. The compounds trichlopyr acid and 3,5,6 trichloro pyridinol were found to be the principal metabolites of Trichlopyr butoxyethyl ester biodegradation within 24 hours. The higher nutrient availability and larger microbial population of the cow dung slurry and soil-pesticide mix was found to affect bioremediation of pesticides under controlled environmental conditions [27]. Research studies showed that adaptability of microorganisms during bioremediation releases enzymes, which metabolizes wide spectrum of anthropogenic chemicals.

The remediation of pesticide residue from soil and water is of prime importance to decontaminate the environment.* Pseudomonas plecoglossicida *is a novel organism for bioremediation of hazardous compounds like cypermethrin [38] and chlorpyrifos (Organophosphate insecticide) by *Pseudomonas aeruginosa *[41]. These microorganisms obtained from cow dung though have the ability for bioremediation in laboratory setups, can also be applied in pesticide contaminated soil and water.

Fenvalerate (a synthetic pyrethroid) is used as a pesticide in agriculture. It has the property to adsorb soil particles and causes contamination leading to the toxicity in soil-water environment. The activated cow dung slurry was used as a source of microbial consortium for bioremediation of fenvalerate amended soil. Fenvalerate was degraded with the formation of prominent intermediates like 4-chloro-alpha benzene acetic acid and 3-phenoxy-benzoic acid over a period of seven days. These intermediates are less toxic than the parent compound and on longer acclimatization in the environment would be mineralized into inorganic biomass and carbon dioxide [42].

Alternatively to the use of pesticides in agriculture, organic farming can be adopted to stop the onslaught of pesticides in the environment. Organic farming usually uses panchgavya—cow dung, cow urine, curd made from cow's milk, cow milk and ghee obtained from cow milk, and other agricultural wastes or locally produced flora on the farm itself. Thus, it is locally available and is a cheap alternative for the improvement in the health of soil and plants alike in agriculture.

## 7. Municipal Sludge with Metals

The effectivity of cow dung compost (earthworm *E. foetida* was used for vermicomposting) upon bioremediation of municipal sludge with heavy metals was seen in Lucknow, India. The various concentrations of leachates exerted moderate to significant inhibition in the plant weight (*Allium cepa*) in a concentration-dependent manner. More than 50% decrease in plant weight in 10% leachate of municipal sludge may be the result of toxicants present in the sludge. The sludge also had many nutritive components along with metals responsible for plant growth, which increased the root weight of A. cepa after vermicomposting. All the metals (Cr, Cu, Ni and Pb) were reduced after vermicomposting. The phytotoxicity and genotoxicity are induced by municipal sludge and can be prevented by vermicompost using cow dung enriched by earthworms [43].

### 7.1. Arsenic

A century-old water purification unit of Faridpur Water Supply (Bangladesh) reduces arsenic contaminated water below Bangladesh standard simply by sunlight and filtration. During 1998 and 1999, experimental studies were carried out in Bangladesh simply using sun, air, iron clay pots (if necessary), and sand filter. The effluent of acid rinses, is mixed with the caustic rinses and this mixed arsenic waste can then be disposed on a prepared bed of cowdung in a shallow pit in earth. The microorganisms in cowdung transform the arsenic to gaseous arsine. This century-old water supply system reduces arsenic concentration from 220 *μ*g/L to 42 *μ*g/L [44].

### 7.2. Oil Spillage

When a solution of cow dung is sprinkled over oil spillage in oceans, it has the capacity to soak the oil. Naturally occurring bacteria in cow dung have the capability to degrade crude oil into simple and harmless compounds. Thus, making the oceans pollution free from the onslaught of man-made disasters and maintaining the aquatic health of oceans.

In a study, cow dung microflora was assessed for aerobic heterotrophic bacteria and petroleum-utilizing bacteria as well as the degradation potential of petroleum-utilizing bacterial isolates. *Acinetobacter* sp., *Bacillus* sp., *Pseudomonas* sp.,* Alcaligenes* spp., and *Serratia* spp. were found as aerobic heterotrophs, and* Pseudomonas* spp. and *Bacillus* spp. were identified as petroleum utilizers in cow dung. Petroleum utilizers in total aerobic heterotrophs ranged from 6.38% to 20% [45].

### 7.3. Petrochemical Industry and Chemical Industry

Bioremediation technology will be useful to the petrochemical industry and chemical industry which generate the waste-containing compounds such as phenol and benzene.

### 7.4. Benzene

Benzene is not biodegradable, is carcinogenic, and is not bactericidal in nature [46]. Bioremediation of benzene can be brought about by using cow dung microflora in a two-phase partitioning bioreactor even at higher concentrations (5000 mg/L). Higher concentration (500 mg/L) of benzene is inhibitory for bioremediation. The *Pseudomonas putida* was isolated from cow dung microflora as a potential benzene degrader and its ability to degrade benzene at various concentrations was seen as 100%, 81%, and 65% degradation at the concentrations of 50 mg/L, 100 mg/L, 250 mg/L within 24 h, 96 h, and 168 h time period respectively [47].

### 7.5. Phenol

The cow dung slurry containing bacteria, fungi, and actinomycetes can be effectively used in degrading phenol ranging from 100 to 1000 mg/L. It is useful to treat the waste containing phenol and to convert the toxicant into nutrient, biomass, and CO_2_ via biodegradation through their intermediates. The experimental findings indicated that when phenol was acted upon by cow dung slurry, and the degradation of phenol began immediately with no lag phase as shown in Table 4 [48].

The *Pseudomonas putida* IFO 14671 has been isolated, cultured, and identified from the cow dung microbial consortium as a high-potential phenol degrader [49].

## 8. Conclusion

Taking cue from Ayurveda, Gomeya/cow dung acts as an excellent bioremediation method. It is cheap, a economically viable option and is locally available in the rural areas of India. Much more exhaustive studies are required to bioremediate the active pharmaceutical agents especially the ones which are nonbiodegradable and persistors in nature. Thus, the adverse effects of these chemicals on flora and fauna can be minimized for a healthy and safe future. These effects can be further studied and validated as per modern research methodology.

## Figures and Tables

**Table 1:** Effect of some synthetic active pharmaceutical ingredients on humans and fauna.

Active pharmaceutical ingredient	Known pharmacology in target organisms	Potential harmful effects on nontarget organisms
Fluoxetine	Sexual dysfunction in human as a side effect	Alters estradiol levels in fish
NSAIDs like diclofenac	Renal toxicity in human	Renal impairment in fish and birds and visceral gout and death of vultures
Ethinyl estradiol	Feminization of males as a side effect	Affecting fertility and development of fish, reptiles, and aquatic invertebrates
Cytotoxics	Wanted effect–anticancer	Reproductive toxicants and cytotoxic to fish and other aquatic species
Enrofloxacin and other antibiotics	“Growth promoters” in agriculture and poultry	Emergence of multidrug resistant strains of pathogenic organisms to humans
Chlorpyrifos Atrazine	Pesticide	Increased susceptibility to *Ambystoma tigrinum* viral infection and increased larval mortality

**Table 2:** Comparison between Gomeya (cow dung) of Indian indigenous cow and cross bred cow.

Contents	Percentage
Organic matter	Similar
Nitrogen	Similar
Manganese	Similar
Calcium	10.8% higher in Indigenous cow
Phosphorus	8.0% higher in Indigenous cow
Zinc	84.1% higher in Indigenous cow
Copper	21.7% higher in Indigenous cow

**Table 3:** Degradation of antimicrobial agents in manure (source of manure including breed of animal not mentioned).

	Manure	% Degradation	Time for degradation (days)	Reference
Chlortetracycline	Cattle	24	84	Runsey et al. [33]

Sulfadiazine	Not mentioned			Ingerslev and Halling-Sorensen [34]

Erythromycin		25	30	Gavalchin and Katz [35]
Streptomycin		0	30	
Penicillin		0	30	
Bacitracin		33	30	

Enrofloxacin	Cattle			Wetzstein et al. [36]

Cyclosporin A		50	60	Thiele Bruhn [29]

**Table 4:** Degradation of phenol with cow dung slurry.

Concentration of phenol	Degradation %	Within time period of
100 mg/L	98.59	24 hrs
250 mg/L	99.4	72 hrs
500 mg/L	99.6	96 hrs
1000 mg/L	Not degraded	Upto 168 hrs

## References

[1] Gualtero SM Pollution Prevention Measures for Unwanted Pharmaceuticals. Industrial Ecology. 2.http://www.seas.columbia.edu/earth/wtert/sofos/Gualtero_IETerm_.pdf.

[2] Kolpin DW, Furlong ET, Meyer MT (2002). Pharmaceuticals, hormones, and other organic wastewater contaminants in U.S. streams, 1999-2000: a national reconnaissance. *Environmental Science and Technology*.

[4] Kobayashi Y, Yasojima M, Komori K, Suzuki Y, Tanaka H (2006). Removal characteristics of human antibiotics during wastewater treatment in Japan. *Water Practice & Technology*.

[5] Lee YJ, Lee SE, Lee DS, Kim YH (2008). Risk assessment of human antibiotics in Korean aquatic environment. *Environmental Toxicology and Pharmacology*.

[6] Carlsson C, Johnsson AK, Alvan G, Bergman K, Kuhler T (2006). Are pharmaceuticals potent environmental pollutants? Part I: environmental risk assessments of selective active pharmaceutical ingredients. *Science of Total Environment*.

[7] ERAVMIS http://www.silsoe.cranfield.ac.uk/ecochemistry/eravmis/.

[8] Heberer T (2002). Occurrence, fate, and removal of pharmaceutical residues in the aquatic environment: a review of recent research data. *Toxicology Letters*.

[9] Hirsch R, Ternes T, Haberer K, Kratz KL (1999). Occurrence of antibiotics in the aquatic environment. *Science of the Total Environment*.

[10] Jones OA, Lester JN, Voulvoulis N (2005). Pharmaceuticals: a threat to drinking water?. *Trends in Biotechnology*.

[11] Ternes TA (1998). Occurrence of drugs in German sewage treatment plants and rivers. *Water Research*.

[12] Marinovich M, Ghilardi F, Galli CL (1996). Effect of pesticide mixtures on in vitro nervous cells: comparison with single pesticides. *Toxicology*.

[13] Thompson HM (1996). Interactions between pesticides; a review of reported effects and their implications for wildlife risk assessment. *Ecotoxicology*.

[14] Thorpe KL, Hutchinson TH, Hetheridge MJ, Scholze M, Sumpter JP, Tyler CR (2001). Assessing the interactive effects of binary mixtures of environmental oestrogens in rainbow trout (Oncorhynchus mykiss) using vitellogenin induction. *Environmental Science and Technology*.

[15] Porter WP, Jaeger JW, Carlson IH (1999). Endocrine, immune, and behavioral effects of aldicarb (carbamate), atrazine (triazine) and nitrate (fertilizer) mixtures at groundwater concentrations. *Toxicology and Industrial Health*.

[16] Seiler JP (2002). Pharmacodynamic activity of drugs and ecotoxicology—can the two be connected?. *Toxicology Letters*.

[18] Randhawa GK, Chanana A, Kullar JS (2009). Ecopharmacology and its future forensic implications—an emerging science. *Medico-Legal Update*.

[19] Andreozzi R, Marotta R, Pinto G, Pollio A (2002). Carbamazepine in water: persistence in the environment, ozonation treatment and preliminary assessment on algal toxicity. *Water Research*.

[20] Daughton CG, Ternes TA (1999). Pharmaceuticals and personal care products in the environment: agents of subtle change?. *Environmental Health Perspectives*.

[21] Zuccato http://www.kfd.org.tr/files/Ekofarmakovigilans.ppt.

[22] Ogden R, Adams DA (1989). Recombinant DNA technology: applications. *Carolina Tips*.

[23] S. Samhita

[24] Ank KG (1995). *Gobar ek jeevanupyogi vastu*.

[26] Nene YL (1999). Utilizing traditional knowledge in agriculture. *Traditional Knowledge system of India and Sri Lanka*.

[27] Geetha M, Fulekar MH (2008). Bioremediation of pesticides in surface soil treatment unit using microbial consortia. *African Journal of Environmental Science and Technology*.

[28] Garg AK, Mudgal V (2007). Organic and mineral composition of *Gomeya* (cow dung) from Desi and crossbred cows—a comparative study. *International Journal of Cow Science*.

[29] Thiele-Bruhn S (2003). Pharmaceutical antibiotic compounds in soils—a review. *Journal of Plant Nutrition and Soil Science*.

[30] Wicklow DT, Carrol GC, Wicklow DT (1992). The coprophilous fungal community: an experimental system. *The Fungal Community. Its Organisation and Role in the Ecosystem*.

[31] Wicklow DT, Detroy RW, Jessee BA (1980). Decomposition of lignocellulose by Cyathus stercoreus (Schw.) de Toni NRRL 6473, a “white rot” fungus from cattle dung. *Applied and Environmental Microbiology*.

[32] Freer SN, Detroy RW (1982). Biological delignification of C-labeled lignocelluloses by basidiomycetes: degradation and solubilization of the lignin and cellulose components. *Mycologia*.

[33] Runsey TS, Miller RW, Dinius DA (1977). Residue content of beeflot manure after feeding diethylstilbestrol, chlortetracycline and Ronnel and the use of Stirofos to reduce population of fly larvae in feedlot manure. *Archives of Environmental Contamination and Toxicology*.

[34] Ingerslev F, Halling-Sorensen B (2000). Biodegradability properties of sulfonamides in activated sludge. *Environmental Toxicology and Chemistry*.

[35] Gavalchin J, Katz SE (1994). The persistence of fecal-borne antibiotics in soil. *Journal of Association of Official Analytical Chemist*.

[36] Wetzstein HG, Schmeer N, Karl W (1997). Degradation of the fluoroquinolone enrofloxacin by the brown rot fungus Gloeophyllum striatum: identification of metabolites. *Applied and Environmental Microbiology*.

[37] Pandey A, Gundevia HS (2008). Role of the fungus—*Periconiella* sp. in destruction of biomedical waste. *Journal of Environmental Science and Engineering*.

[38] Boricha H, Fulekar MH (2009). *Pseudomonas plecoglossicida* as a novel organism for the bioremediation of cypermethrin. *Biology and Medicine*.

[39] World Health Organization Report on TBEE. Environmental Health Criteria.

[40] Chauhan RS, Singhal L (2006). Harmful effects of pesticides and their control through cowpathy. *International Journal of Cow Science*.

[41] Fulekar MH, Geetha M (2008). Bioremediation of Chlorpyrifos by Pseudomonas aeruginosa using scale up technique. *Journal of Applied Biosciences*.

[42] Geetha M, Fulekar MH (2010). A remediation technique for removal of fenvalerate from contaminated soil. *Asian Journal of Water, Environment and Pollution*.

[43] Srivastava R, Kumar D, Gupta SK (2005). Bioremediation of municipal sludge by vermitechnology and toxicity assessment by Allium cepa. *Bioresource Technology*.

[44] http://www.sos-arsenic.net/.

[45] Akinde SB, Obire O (2008). Aerobic heterotrophic bacteria and petroleum-utilizing bacteria from cow dung and poultry manure. *World Journal of Microbiology and Biotechnology*.

[46] Hardman JG, Limbird LE, Gilman AG (2001). *Goodman’s and Gilman’s The Pharmacological Basis of Therapeutics*.

[47] Singh D, Fulekar MH (2010). Benzene bioremediation using cow dung microflora in two phase partitioning bioreactor. *Journal of Hazardous Materials*.

[48] Singh D, Fulekar MH (2007). Bioremediation of phenol using microbial consortium in bioreactor. *Innovative Romanian Food Biotechnology*.

[49] Singh D, Fulekar MH (2009). Bioremediation of phenol by a novel partitioning bioreactor using cow dung microbial consortia. *Biotechnology Journal*.

